# In‐house auto cutoff sensor device for radiotherapy machine to monitor patient movements

**DOI:** 10.1120/jacmp.v9i3.2800

**Published:** 2008-06-23

**Authors:** S Senthilkumar, V Ramakrishnan

**Affiliations:** ^1^ Dept. of Radiotherapy Government Rajaji Hospital & Madurai Medical College Madurai India; ^2^ Dept. of Laser Studies Madurai Kamaraj University Madurai India

**Keywords:** Radiotherapy, sensor device, cancer, IR rays, PMMD

## Abstract

Radiotherapy is an effective treatment method for cancers. During radiation treatment, a patient must be in the same position from the start to the end of radiation treatment. Patient movements are usually monitored by the radiation technologists through the closed circuit television (CCTV) during treatment. If the patient makes a small movement, it is difficult to be noticed by them. In the present work, a simple patient movement monitoring device (PMMD) is fabricated to monitor the patient movement. It uses an electronic sensing device. It continuously monitors the patient's position while the radiation treatment is in process. The device has been retrospectively tested on 86 patients whose movement and distance were measured. The results show that 24 patients moved 1 cm to 2.5 cm from their initial position during the external beam radiotherapy (EBRT). Hence, the device can potentially be used to control and monitor patient movement during EBRT. In addition, an audible alarm situated at the control panel of the treatment room is provided with this device to alert the radiation technologists. It is an inexpensive, compact device which can be used in any radiotherapy machine. It can prevent patients from being treated in a wrong position and therefore improve the quality of the radiation treatment.

PACS Number: 87.53Dq

## I. INTRODUCTION

The success of radiotherapy is to deliver maximum dose of radiation to the tumor tissue. At the same time, the minimum dose of radiation has to be ensured to the surrounding normal tissues to reduce the incidence of radiation of acute and late effects.^1^, ^2^ To achieve this, proper treatment planning and careful execution is mandatory. Patient immobilization is an important parameter during the radiotherapy treatment. There are different kinds of immobilization devices used to immobilize the patient for radiotherapy treatment in clinical condition. However, immobilization devices are not used for all patients because of the non‐availability of the large thermoplastic sheets for regions like the breast, cervix, extremities etc., and also the skin sparing effect of the acquaplastic thermoplastic sheets. Immobilization devices are often used for the brain, and head and neck cancer patients, but not for patients with lesions in other regions like the esophagus, breast, cervix and extremities. The head and neck immobilization device is not always used in some of the hospitals.

During treatment, if the patient moves away from the initial setup position, it is not possible to achieve the goal of radiotherapy treatment. Patient motion is usually caused by uncontrolled physiological behaviours such as coughing, swallowing, passing gas, or is due to the patient adjusting to a more comfortable position. The patient may not tolerate staying in the same position for a long time. Because patient movements could potentially induce severe systematic position errors, monitoring patient motion during treatment is especially crucial for lengthy treatment procedures.^(3)^

Patient motion can be monitored by the radiation technologist through a video camera. However, the human eye is usually not able to quantify such motion and it is not reliable for detecting slow position changes during the whole course of treatment. Infrared (IR) cameras have been used to quantitatively monitor the respiratory motion of a patient, with infrared external markers placed on the patient surface.^4^, ^6^ However, IR cameras used for those studies are expensive. Once, the patient positions for the external beam radiotherapy (EBRT) in the radiotherapy machine are set, they should not move until the treatment is completed. The radiation technologists can monitor patient motion either in front of the video monitor, which is located on the control panel outside the treatment room, or beside the patient in the treatment room. After positioning the radiotherapy machine, the radiation technologists (RT) have to walk out the treatment room through the maze wall, close the main door, and then switch ON the radiotherapy machine. This takes about a minute. During this time the patient's movement will not be monitored by the technologist. Patient movement during this time is not identified, so an incorrect treatment may be delivered to the patient.

In our hospital we treat more then 100 patients per day with only two technologists. Continuously monitoring the patient's movement through the closed circuit television (CCTV) is not possible, because during this time the technologists are performing a number of administrative tasks like entering the patient information in the hospital register. In the mean time, if the patient moves it is not noticed by the technologists. Even though the technologists are continuously monitoring the patient's movements, other patients, their attendees, or other hospital workers, may disturb their attention.

To avoid errors caused by patient movements during radiation treatment, the patient position should remain the same and be monitored continuously. Based on this, we fabricated an auto cutoff sensor device for radiotherapy machines. It is an inexpensive, compact, light‐weight electronic device. This patient movement monitoring device (PMMD) is potentially useful for delivering the correct dose to the tumor area and for avoiding radiation of the normal surrounding area during EBRT for cancer patients.

## II. MATERIALS AND METHODS

### A. Construction of sensor device

**Figure 1:**
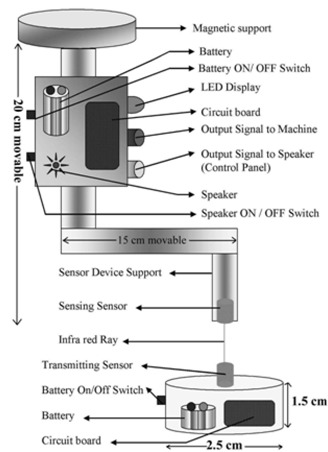
Block diagram of PMMD

**Figure 2:**
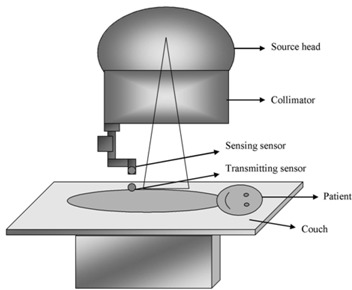
Block diagram of radiotherapy machine with PMMD

The device consists of two main parts. One part is a sensing device and the other is a transmitting device. The sensing device is attached to the collimator of the cobalt‐60 machine with the help of a permanent magnet. It can be attached and detached from the collimator with ease. The length of the sensing device is 20 cm, which can be varied. Figs. 1 and 2 show the block diagram of PMMD and PMMD attached to the radiotherapy machine along with the treatment position of the patient, respectively. This sensing device looks like a mechanical SSD scale. It can be attached to the collimator exactly like the attachment of the mechanical SSD rod with the collimator. The distance of the sensing device is adjustable and it can be rotated at any angle.

### B. Transmitting sensor device

The transmitting device is small and compact; it is 2.5 cm in diameter. The weight of the cylindrical box is 15 gm and the height is 1.5 cm. The power supply of the device is 1.8 voltages. It has its own controlling switch, which controls the power supply to the circuit. The transmitting sensor device can be placed over the patient's body surface with a sticker at the bottom of the transmitting device. This device can be placed on the anterior and lateral surfaces of the body.

### C. Sensing of patient movement

After checking the field size and the SSD, the patient is aligned in the radiotherapy machine for a particular treatment. Finally, the transmitting sensor device is placed near the radiation delivery area. The transmitting device will stick on the patient body, because it has sticking gum at the bottom of the device. After placing the transmitting device on the patient body, the sensor device is attached to the collimator. The transmitting device emits infrared rays. The sensor device is aligned parallel to the infrared rays. When the transmitting device and the sensor device are aligned along the axis, there will be no signal to the output devices.

### D. Output devices

There are two kinds of output devices which are used in the sensing device: audible alarm and breaking circuit. Audible alarm is a speaker device. It is situated in the control panel of the radiotherapy room. The speaker is connected directly to the output signal of the sensing device through electrical wire. Breaking circuit is a single phase controller circuit. The output of the sensor device can be connected directly by electrical circuit in series to the radiotherapy machine. Any one of the above output devices can function during the radiotherapy treatment.

### E. Patient movements during the treatment

If the patient moves during the treatment, the device will alert the technologists to interrupt the treatment. If the circuit is connected with the radiotherapy machine's electrical supply, it will turn the machine to standby mode. The process is as follows. When the patient moves during EBRT, the transmitting device, which is placed over the patient, will move. The sensor will not get the transmitting signal from the transmitting sensor device. If there is no signal to the sensor from transmitting device, it will allow the negative voltage to the circuit from the transistor. Then the circuit gives the signal to the alarm circuit, as well as to the breaking circuit. Since the output signal is connected with the speaker it will raise the audible sound or if it is connected with the breaking circuit it will turn the machine off. As a result, the radiation machine will stop emitting the radiation. If the output is connected to both the processes, they will function simultaneously unless the transmitting device and sensor device are aligned in parallel. Hence, it is not possible to stop the audible sound as well as restart the radiotherapy machine before the device gets aligned.

### F. Construction of the photosensor circuit

It is an electronic‐controlling device which monitors the patient movements with the help of a 10 cmx5 cm PCB circuit board. This circuit functions with the help of different electronic components such as a battery, switch, LED, speaker, photodiode, resistance, transistor etc., to give the electric potential to the sensor circuit. A switch is placed between the circuit and the battery. When the switch is in OFF condition there will be no power supply to the circuit and the sensor will not function. When the two sensors coincide, the LED glows. The speaker is an audible device which produces a sound when the patient moves. Photodiode is used to continuously sense the phototransistor signal. If there is no communication between the photodiode and the phototransistor due to the movement of the patient's body, the electricity in the circuit will be cutoff. The function of the resistor is to minimize the power supply to the circuit and to control the overflow of electrons in the circuit. A BC 547 transistor serves different purposes in the circuit. When the base of the transistor receives the negative voltage, the collector will give the positive voltage to the circuit and the emitter is grounded. It will cut off the electrical signal depending upon the function of the circuit. The phototransistor transmits the infrared waves. If the transmitting device moves away from the path of the sensing device, the device will break the power supply of the radiotherapy machine.

### G. Construction of photo transistor circuit

The phototransistor circuit consists of a battery supply, an ON/OFF switch, a resistance and a phototransistor. It is small and can be placed over the patient's body to give information about the patient's position to the sensor. Two batteries (each 1.5 V) are used and give power supply to the phototransistor circuit. The model of the batteries are AA. The switch is placed between the circuit and the battery. When the switch is in OFF condition there will be no power supply to the circuit and therefore the sensor will not function.

### H. The sensing device

The sensing device is attached to the collimator. It is made up of an acrylic rod. The upper part of the rod is fixed with a magnet. The attaching device consists of a vertical rod. The lower part of the vertical rod is L‐shaped and it is connected with movable acrylic rod. The sensor device and circuit are fixed in the vertical rod. The sensing photodiode is kept at the lower end of the L‐shaped acrylic rod. The sensor is connected with the circuit through electrical wires. The L‐shaped acrylic rod is movable in X and Y directions and also rotates horizontally about 360°. A permanent magnet is used to attach the sensing device to the radiotherapy machine. The L‐shaped movable acrylic rod helps make the correct position of the sensor diode with the transmitting devices and along the axis.

### I. Design of the circuit

The circuit consists of four transistors, four 1 KΩ resistors, four 10 KΩ resistors, a photodiode, a phototransistor, a LED and a switch. The transistors are connected in parallel. The collector terminal of one transistor is connected with the base terminal of another transistor. 1 KΩ resistor is connected in series to the base terminals of the four transistors respectively. Ten KΩ resistors are connected in parallel to the collector terminals of the four transistors respectively. The LED is connected in series with the second transistor collector terminal and the switch connects the second and the third transistor to the entire circuit.

### J. Working principle

The function of the sensor circuit is based on the principle of infrared waves passing through the phototransistor. When the phototransistor carries negative voltage to the base terminal of the first transistor, the base terminal gives negative voltage to the collector terminal. Consequently, it gives negative voltage to the second transistor's base terminal and hence to its collector. This turns the LED to ON condition which indicates that the IR is in correct position. If the sensor is moved, then the IR rays deviate from its axis. So the third transistor and the fourth transistor's bases collect negative voltage from the emitter terminals. During this period, the buzzer is in the ON condition and also at the same time the LED turns to OFF condition. When the transmitting and the sensing device are coincident, the buzzer is in OFF condition. This circuit requires 9 to 12 volts. It also consists of two devices; one is a sensing device and the other is a transmitting device. Both of these devices function separately with their own battery power supply. The sensing device consists of a 9 volt battery, an ON/OFF switch, a LED, transistors, speakers, resistors and sensors which are the main components of these circuits.

## III. RESULTS AND DISCUSSION

### A. Phantom study

To measure the sensitivity and tolerance limit of the device, a phantom study was carried out in the +X, −X, +Y and −Y directions. The sensitivity of the device at various positions in the phantom was measured and the results are presented in Table 1. During the measurement, it was observed that the device was triggered and the radiotherapy machine went into standby mode when the treatment table was moved off 0.5 cm from the center of the device in the +X, −X, +Y and −Y directions with a fixed sensitive area of 1 cm. Similarly, the measurements were repeated and observed for the other fixed sensitive areas like 1.5, 2.0 and 2.5 cm. The tolerance limits of the device for allowed values and to stop the treatment was below 0.5 cm and above 0.5 cm, respectively.

**Table 1:** Sensitivity measurements of PMMD at various positions

*Device position in phantom*	*Sensitive area fixed in phantom (cm)*	*Direction of treatment table movement for trigger (cm)*			
		+X	−X	+Y	−Y
Position I	1	0.5	0.5	0.5	0.5
Position II	1.5	0.75	0.75	0.75	0.75
Position III	2	1	1	1	1
Position IV	2.5	1.25	1.25	1.25	1.25

### B. Patient movement monitoring study

**Figure 3:**
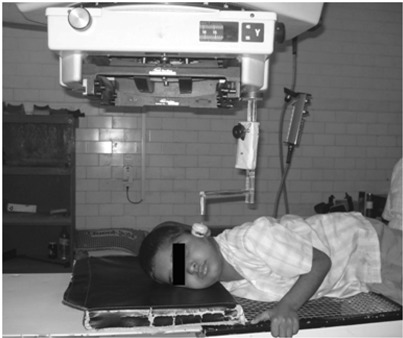
Clinical photograph of PMMD

Fig. 3 shows the brain tumor of a young boy in the clinical treatment position. The PMMD is used with the radiotherapy machine. This equipment is utilized for observing the movement of 86 patients. Of those patients, 40 had cervical cancer, 10 had breast cancer, 8 had head and neck cancer, 5 had lung cancer, 10 had esophagus cancer, 10 had brain tumors, 2 were pediatric patients and 1 was unconscious. It is clearly observed in Table 2 that among the 86 patients, 24 (2 cervix cancer, 3 breast cancer, 8 head and neck cancer, 3 lung cancer, 1 esophagus cancer, 4 brain tumor, 2 pediatric and 1 unconscious) moved from their position during the treatment, whereas the rest received the radiation without movements. The patient movement sensing system output was connected with the radiotherapy equipment circuit, which was connected near the interlocking door circuit in series connection. When the sensing system detected the movements of the patient, the result cut OFF the electrical power supply in the radiotherapy machine. Thereby, the machine stopped emitting radiation. When we entered the treatment room and checked the patient position, the patient had moved from 1 to 2.5 cm laterally from the actual planned field area. The patient was repositioned exactly to the previous treatment position and after that the machine was switched ON for radiation exposure. It is possible to restart the machine only after repositioning the patient. After proper instructions were given and more care taken, the frequencies of motion of these patients were controlled. A large number of interruptions were found in the head and neck, pediatric and the unconscious patients. Therefore it would be more effective to sense the patient's movement during the radiotherapy treatment in two ways; one is to turn the radiotherapy machine in standby mode and the second is to alert the radiation technologist by audio speaker.

**Table 2:** Patient movements for different types of cancer

*Types of cancer*	*Number of patients*			*Distance moved (cm)*	*Reason for patient movements*
	*With movement*	*Without movement*	*Total patients*		
Ca. Cervix	2	38	40	1	Adjusting for comfortable position
Ca. Breast	3	7	10	1.5	Pain of hand
Head & Neck	8	0	8	1.5	Neck pain/Swallow
Ca. Lung	3	2	5	1	Cough
Ca. Esophagus	1	9	10	1	Cough
Brain tumor	4	6	10	1	Pain
Pediatric	2	0	2	2	Child behavior
Unconscious	1	0	1	2.5	Unconsciousness

## IV. CONCLUSION

A patient movement monitoring device was successfully fabricated and patient movements were quantitatively analyzed using this device. The PMMD is an electronic device that is compact, small, ligh‐weight, inexpensive and easy to handle. The sensitivity of this device can be varied for different cancer sites in the human body. When the patient moves during radiotherapy treatment it turns the electrical power supply off. Hence, the radiation is stopped. Therefore it can prevent unnecessary radiation exposure to the patient's normal tissues and can also help deliver the radiation to the correct tumor location. Another method to control the motion is to use an audible device. With this method, when the patient moves from the radiation area the alarm will raise its sound. The raising sound alerts the radiation technologists. Using this alarm system, the patient can be repositioned after manually interrupting the treatment machine. It was also found that the breathing motion of the patient did not interrupt the PMMD device. Studies were conducted on 86 patients with different types of cancers. The results demonstrated that the device was able to detect patient movements with a sensitivity of about 0.5 cm. This device can potentially be used to monitor and control patient movement during EBRT.

## ACKNOWLEDGEMENTS

One of the authors (SSK) would like to thank the Dean, Dr. S. Vasanthamalai, and Dr. J. Jebasingh, Radiation Oncologist, Department of Radiotherapy, Government Rajaji Hospital & Madurai Medical College, Madurai, Tamilnadu, India and N. Ponnusamy, Dept. of Physics, Kalasilingam University, Kirishnankovil, India for their help to develop this device.
